# University-Based Researchers as Knowledge Brokers for Climate Policies and Action

**DOI:** 10.1057/s41287-022-00526-0

**Published:** 2022-05-17

**Authors:** David Lewis, M. Feisal Rahman, Revocatus Twinomuhangi, Shababa Haque, Nazmul Huq, Saleemul Huq, Lars Ribbe, Asif Ishtiaque

**Affiliations:** 1 London School of Economics & Political Science, London, UK; 2 International Centre for Climate Change and Development (ICCCAD), Independent University of Bangladesh, Dhaka, Bangladesh; 3 Makerere University Centre for Climate Change Research and Innovations (MUCCRI), Makerere University, Kampala, Uganda; 4 ICLEI, Bonn, Germany; 5 ITT, TH Köln, Cologne, Germany; 6 School for Environment and Sustainability, University of Michigan, Ann Arbor, USA

**Keywords:** Climate change, Knowledge brokers, Research-policy interface, Evidence-informed decision-making, Evidence-to-policy, University-based research

## Abstract

Responding effectively to climate crisis requires strong science-policy links to be put in place. Past research on the research-policy interface indicates longstanding challenges that have become more acute in the case of climate science, since this requires multi-disciplinary approaches and faces distinctive political challenges in linking knowledge with policy. What can be learned from the experiences of university-based researchers seeking to influence policy as they try to operate in the brokering space? With this in mind, an empirical study was designed to capture the detailed views and experiences of forty researchers in four universities across four countries—Bangladesh, Germany, Uganda and UK. It found a wide range of different researcher attitudes to policy engagement, diverse methods of engaging, a preference for working with government and civil society over private sector policy actors, and a perceived need for more university support. The findings suggest a need to rethink conditions for engagement to create spaces for knowledge exchange and cooperation that can contribute to policies for societal transformation. More attention also needs to be paid to interdisciplinary research approaches, improving research connections with private sector actors, and strengthening university research links with local communities. Finally, the position of university based researchers in the Global South will require strengthening to improve North–South knowledge exchange, capacity development, and incentives for policy engagement.

## Résumé

Afin de répondre efficacement à la crise climatique, il est nécessaire de mettre en place des liens solides entre la science et la politique. Les recherches antérieures sur l'interface recherche-politique indiquent des défis de longue date qui sont devenus plus aigus en ce qui concerne la science du climat, car cela nécessite d’adopter des approches pluridisciplinaires et de faire face à des défis politiques bien distincts pour faire le lien entre les connaissances et les politiques. Que peut-on apprendre des expériences des chercheurs et chercheuses universitaires qui cherchent à influencer les politiques tout en essayant d’entrer en négociation ? Dans cette optique, une étude empirique a été conçue pour recueillir les points de vue et les expériences détaillés de quarante chercheur·euse·s de quatre universités réparties dans quatre pays - le Bangladesh, l'Allemagne, l'Ouganda et le Royaume-Uni. L’étude a détecté un large éventail d'attitudes différentes des chercheur·euse·s à l'égard de l'engagement politique, diverses méthodes d'engagement, une préférence pour le travail avec le gouvernement et la société civile par rapport aux acteurs politiques du secteur privé, et un besoin perçu de plus de soutien universitaire. Les résultats suggèrent la nécessité de repenser les conditions d'engagement afin de créer des espaces d'échange de connaissances et de coopération qui peuvent contribuer aux politiques de transformation sociétale. Il faut également accorder une plus grande attention aux approches de recherche interdisciplinaires, à l'amélioration des liens entre la recherche et les acteurs du secteur privé et au renforcement des liens entre la recherche universitaire et les communautés locales. Enfin, la position des chercheur·euse·s universitaires dans les pays du Sud devra être renforcée pour améliorer l'échange de connaissances Nord-Sud, le développement des capacités et la motivation à l'engagement politique.

## Introduction

The challenge of tackling the ‘super wicked problem’ of climate crisis remains urgent and complex, because the central authority needed to address the problem of climate is non-existent or weak, because those who are trying to find solutions have most likely also contributed to the problem, and because the time needed to take meaningful action is rapidly running out (Levin et al. 2012). New, carefully developed, and well-informed policies across a wide range of areas will be required. In moving towards solutions, there is an increased recognition that more inclusive multi-actor policy and governance arrangements are also needed, and that universities will have important roles to play within such arrangements. In building these pluralist approaches to policy a key challenge is to improve the relationship between researchers and policy makers.

In this article we explore the experiences of university-based researchers who are trying to engage with the world of climate policy using their findings and ideas, sometimes working in what are described as ‘knowledge broker’ roles. The paper analyses their views and experiences, asks how their efforts can be better understood, and concludes with some practical ideas about how such work can be better supported. These conclusions are placed within the wider context of debates around improving the overall relevance of university-based research.

We are not naive about the strong interests and inequalities of power that constrain efforts to link climate research and policy action in more productive ways, nor about the short-termism displayed by elected politicians, particularly in the Global North, that leads them to resist taking any action on climate issues. Recently a group of leading scientists went as far as to suggest as one possible response to this problem should be a moratorium on climate change research and further IPCC assessments ‘until governments are willing to fulfil their responsibilities in good faith’, arguing that the implicit social contract between science and society is ‘broken’ (Glavovic et al. 2021)^1^. We also recognise that funding biases, and the deliberate efforts of the fossil fuel industry, continue to obstruct and deflect research findings from reaching the public sphere and informing policy action (Brulle 2014; Betts 2021). However, we do not see any alternative to continuing the search for productive ways to rethink and act on these issues.

### Framing Relationships Between Research and Policy

A large body of scholarly work in the social sciences asserts the desirability of improving the science-policy interface. Mainstream views of the relationship tend to follow the ‘policy process’ literature that reflects a rational choice approach to policy and puts government decision makers at the centre. The policy process is usually understood in terms of a set of linear decision making ‘stages’ such as problem identification, policy formulation, policy implementation and evaluation (e.g. Bulmer 1986), as a ‘policy cycle’ (Lasswell 1956), or as a ‘policy chain’ (Zezza and Llambi 2002). Within this type of thinking, researchers are encouraged to seek opportunities to intervene within these different stages of the policy process in order to try to influence government decision makers with their findings, often by drawing on specialised tools for policy engagement such as writing ‘policy brief’ summaries of findings and implications or making presentations at specially convened ‘round table’ meetings.

By contrast, a less technical and more anthropologically informed approach to understanding the workings of policy processes work draws on the broader idea of ‘policy worlds’ (Shore and Wright 2011). This is a critical perspective that mainly takes an *interactive* rather than a *linear* view of policy, pays close attention to the workings of power relations and special interests, and offers a pluralistic view of the actors involved in policy alongside the government—such as business, civil society organizations, and community groups and movements. Indeed, the importance of fostering participation by non-state actors in global environmental governance is one that has been increasingly highlighted (Nasiritousi et al. 2016). At the same time, without recognising the damaging effects of lobbying and more covert forms of action by special interest groups such as the fossil fuel lobby we cannot fully understand how policy worlds operate.

Approaching policy in terms of ‘knowledge, actors and spaces’ (McGee 2004) offers the potential to analyse complex worlds of policy in more realistic and nuanced ways. It draws attention to the different levels of policy action, the diverse arenas in which various policy actors interact, the issue of who is or is not included in policy processes, the micro-politics of how knowledge is transformed into ‘evidence’ and makes clear that policy and action always emerge from competing knowledge claims and contestations. The question of who participates and on what terms, is particularly crucial.^2^ Finally, the approach highlights the importance (and desirability) of ‘co-production’ via collaboration between knowledge producers and knowledge users that can bring about effective change (Lemos et al. 2018; Jasanoff 2004; Mitlin et al. 2019). These interactive and co-production perspectives open up more imaginative space for thinking about the importance of intermediary roles alongside those of researchers and decision makers, including the idea of ‘knowledge brokerage’ work.

The concept of the ‘knowledge broker’—a role that can be defined as ‘the dissembling and reassembling of extant ideas, artifacts, and people’ (Hargadon 2002)—is potentially helpful because it moves us beyond simplistic and highly technical ‘bridge the gap’ perspectives. For example, actor-network theory (ANT) challenges the idea of research and policy as two distinct bounded organisational worlds and instead invites us to recognise their instability and examine the informal networks and processes connecting their meanings and practices. Using this frame Cvitanovic and Hobday (2018, p. 1) argue that decision-makers may for example draw more heavily on types of ‘experiential knowledge’ than on evidence-based science. They are critical of the idea of a ‘science policy gap’ because of the way it ‘validates the misleading and outdated notion that scientists and decision-makers are distinct groups of individuals divided by a range of unsurmountable cultural and epistemological differences, rather than recognising their interdependency and shared values.’

Instead of just thinking about how researchers need to better communicate their results *to* policy makers, we also need to consider how decision makers *and* researchers construct knowledge that can help devise new solutions to pressing problems. Brokers also engage in forms of translation that have both performative and representational dimensions, contributing to the shaping of social worlds and professional identities, and requiring us to move beyond merely functional perspectives implied by linear models of policy (Mosse and Lewis 2006).

We therefore need to treat taken for granted categories such as ‘research’ and ‘policy’ with caution. Rather than simply seeing them as separate worlds that should be better connected, we should start by understanding how they are already entangled, while recognising the fact that these are unstable categories that are subject to change. For example, the idea of ‘boundary-work’ usefully expresses this idea that the separation of science from policy is never clear cut, with dividing lines that shift and are constantly being drawn and redrawn (Gieryn 1983). The work of the knowledge broker role is typically complex and varied too, including ‘managing knowledge’ (e.g. creating, translating and applying it), ‘linkage and exchange activities’ (e.g. enhancing relationships between knowledge creators and users and maintaining their identities) and ‘capacity building’ (e.g. building skills and capacities to generate, understand, and apply knowledge) (Bornbaum et al. 2015).

### Climate Research and the University Sector

Scientific capacity is critical to the process of generating ‘relevant’ knowledge that can be drawn upon in the formulation and implementation of effective policy to address climate crisis (HoLem et al. 2011). However, even a cursory engagement with the political economy of knowledge production will tell us that a significant North–South divide exists in the generation and application of knowledge around climate issues. For example, an analysis of over 1500 climate change publications between 1990 and 2010 revealed that ‘the case country publication bias is towards richer, cooler and less vulnerable countries, with high carbon emissions, with stronger institutions and more press freedom’, and that even when focused on the most vulnerable countries, it remains dominated by Global North researchers rather than those who are locally based (Pasgaard et al. 2015). There is also evidence of publication bias in the production of the Intergovernmental Panel on Climate Change (IPCC) reports (Hulme and Mahony 2010; Vardy et al. 2017; Thomas 2018).

Article 11 of the 2015 Paris Agreement identifies ‘capacity building’ as fundamental for achieving its goals, and universities are acknowledged as key partners in building long-term capacities, particularly in those countries most vulnerable to climate change (Nasir et al. 2017). Yet there appears to have been little progress to date in addressing these capacity issues (Nakhooda 2015). When it comes to climate knowledge, there is a disconnect therefore between knowledge supply and capacity needs, and this has particularly negative implications in relation to efforts to integrate ‘indigenous’ and locally-generated knowledge into policy and planning processes (Pasgaard et al. 2015).

At the same time, the roles of public and private universities in both the Global North and South are currently coming under greater scrutiny. Universities everywhere face changing expectations about their purposes and increasingly operate in more constrained resource environments. The balance between universities’ role in producing both private and public goods, in terms of individual benefits to graduates and gains to society as a whole, is also increasingly a matter for debate. For example, the journal *Nature* reports that populist political parties in the Netherlands, Italy and Spain have pushed a view that universities are increasingly ‘elitist and irrelevant to society’ (Witze 2020). In the UK, universities are under increasing pressure to demonstrate their relevance and contribution to society, offer improved ‘value for money’, and provide students with relevant skills and experiences to enter the job market. There are also increasing calls around the world to ‘decolonise’ the university and open up new visions of its purpose, structure and values that can de-centre and challenge dominant Eurocentric academic models (Mbembe 2016). Meanwhile, the role played by university research in the development of Covid-19 vaccines may have provided a countervailing tendency towards the strengthening of positive public opinion in some quarters.

There are also growing pressures from civil society groups, and from within some universities themselves, to strengthening their civic roles and responsibilities, including community engagement. For example, the Talloires Network of Engaged Universities, housed at Tufts University in the US, is a coalition that brings together 417 university leaders in 79 countries committed to strengthening the social responsibilities of their institutions, and it is the largest international network of its kind. There is also a need to make knowledge production and access more democratic. For example, civil society groups such as INASP (originally the International Network for the Availability of Scientific Publications) are campaigning for the creation of more ‘equitable knowledge ecosystems’. There is also pressure from governments and funders to improve the relevance of university research. In the UK, for example, the government’s research excellence framework (REF) includes a component that seeks to measure research impact. This has led to resistance by some academics who see impact as the unwelcome imposition of audit culture, while for others it offers the opportunity to build community partnerships that can challenge the traditional dominance of elite research hierarchies (Macdonald 2018).^3^

The nature of the climate crisis may require nothing less than a complete rethink of the way university-based research is organised. The modern university with its twin focus on producing pure knowledge and training workers for the job market, as Ford (2002) and others have long argued, may be too closely aligned with the destructive nature of modernity to enable the creative rethinking of economy and society in the Anthropocene. Traditional disciplines of knowledge production—whether in the natural sciences, the social sciences or engineering—are patently insufficient for tacking complex, multidimensional problems. For example, the novelist Amitav Ghosh has argued in his book *The Great Derangement* (2016) that the profound nature of climate crisis requires us to produce new ways of thinking that can be driven as much by fiction as by science and politics.

Indeed, climate crisis has been called ‘perhaps the greatest call to interdisciplinary arms’ (Callicott 2010, p. 494). It has the potential to bring disciplines together in ways that challenge conventional assumptions, and in ways that can generate more creative and ‘joined up’ thinking to solve problems. For example, *Ecologist* editor Satish Kumar recently called for universities to build closer links in the teaching of economics and ecology.^4^ Interdisciplinary research is far from easy, or straightforward, given hierarchies of power and knowledge, and the different capacities of disciplines to communicate across the practitioner and policy maker divide, but it can ‘expand the insights’ of the researchers and partners involved (Beall et al. 2019).

### Objectives and Methods

The objectives of the study were to understand how university based researchers approach policy engagement, how they attempt to engage with ‘policy worlds’, and to learn from their experiences in order to develop better strategies for influence. The study was funded by the Rockefeller Foundation and the LSE Institute for Global Affairs (IGA), and interviews took place during 2019–2020. The focus was on researchers who work on climate crisis and environment from a wide range of academic disciplines. Four universities participated: Makerere University, Uganda, the Independent University of Bangladesh (IUB), TH Köln, Germany, and the London School of Economics and Political Science, UK (see Annex). These particular universities were selected because each contains researchers active in local, national and international engagement around climate research. A further objective was to achieve a comparative frame combining engaged researchers in the Global South *and* the Global North, which to the best of our knowledge, has not been attempted before.

**Fig. 1:**
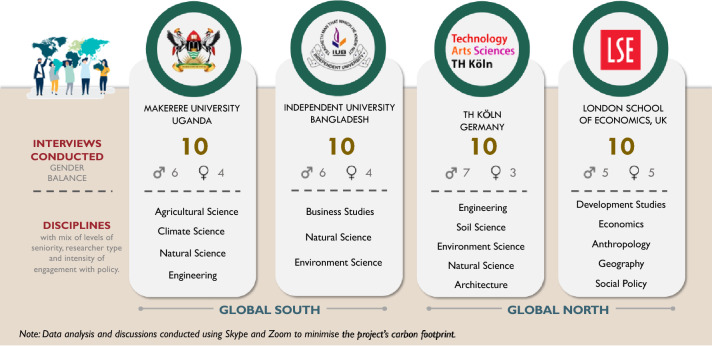
Study design: academic engagement with policy across four universities in the global south & global north

In each institution, a purposive sample of ten researchers was drawn up to access a mix of researchers representing different academic disciplines, levels of seniority, position (permanent teaching faculty and contract researchers) and intensity of engagement with policy (Fig. 1). Data was collected using a basic survey proforma and a set of semi-structured interview probe questions. This gave us a total of 40 interviews undertaken with university based researchers from a wide range of academic disciplines working on climate crisis and environment issues across the four countries. An effort was made to secure a gender balanced sample, though it was not possible to achieve this across all four universities, with the male/female ration 5:5 at LSE, 6:4 at Makerere and IUB, and 7:3 at TH Köln, giving a total of 24 male and 16 female respondents overall. These differences in part reflected the prevailing gender balance among researchers at certain institutions.

Data analysis and discussions of the country level data were conducted among the group of international researchers using Skype and Zoom to minimise the project’s carbon footprint. Interviews were recorded and transcribed. Written informed consent was obtained from each of the participants. The design of the study was reviewed at LSE and complies with the university’s Research Ethics Policy. After the interviews, contact was made with a small sample of policy makers mentioned by our interviewees and a round table meeting organised in each country to reflect on issues raised. This was designed as an exercise to validate our interview data rather than to extend it, but in a few cases new insights were also obtained. In the LSE case the exercise took the form of supplementary individual interviews rather than a round table meeting.

There are two limitations to our design that should be noted. First, our use of purposive sampling means that we are presenting perspectives drawn from our sub-group of interviewees, rather than general conclusions about the situation at each university. Second, we are primarily concerned with researcher modes of engagement in the field of climate research, and not with the content or effects of the research that was undertaken.

## Findings: Multiple Motivations, Diverse Engagements

**Fig. 2:**
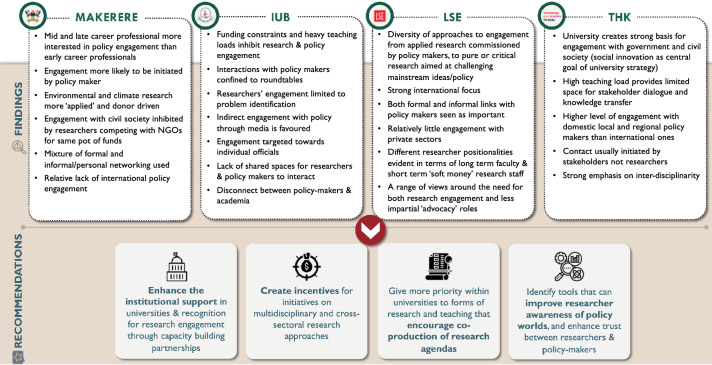
Key findings & recommendations

In this section we first outline general findings before moving on to consider more detailed insights on research-policy engagement (see Fig. 2). Only six interviewees indicated that they had never had any form of interaction with a policy maker at all. Four of these were early career researchers, and the rest were mid-career. The two researchers from IUB who indicated they have never interacted with a policy maker were both female.

When asked at which points in the policy process they had attempted to engage, the majority reported involvement only with ‘problem identification’ and/or ‘agenda setting’, rather than with implementation or evaluation. Five of those interviewed (four of whom were male and from the Global South) indicated they had engaged with all four stages, and a further seven interviewees had engaged with three stages. Each of these eleven were either in their late or mid-career stages, and only three were female. No specific trend around this theme between the Global South and the Global North could be identified.

As to which party had initiated the researcher-policy interaction, the majority of our informants (23) indicated that it had been the policy maker. Among the interviewees only seven researchers indicated that they had judged their engagement with the policy process to be ‘very effective’, while another 21 responded that they had viewed it as only ‘slightly effective’. Those who responded ‘very effective’ were in their late or mid-career and the majority (five) were male.

Around 40% of our interviewees reported some form of engagement with the private sector around their research, a finding that was more or less consistent across all four universities. Given the ambiguity around whether a private foundation or business association should be properly classified as private sector or as civil society, the true figure could be be lower. Contact with civil society organisations was somewhat higher, but the extent varied significantly between the universities, with TH Köln reporting the least level of engagement, perhaps due to its more technical research focus.

### Why Do Researchers Engage?

We found a mix of different attitudes to policy engagement reported among academic researchers. Most felt it was their duty to make sure that their research findings did not remain confined to the so called ‘ivory tower’ of elite academia. They sought to engage, with varying levels of success, using a range of different methods. These included attending invited policy round tables, participating in formal and informal research-policy networks, writing policy briefs, and undertaking consultancy work—building on their research knowledge—that they felt might contribute to real world change. Despite the different orientations of universities, common themes emerged in relation to researcher motivations for policy engagement.

Chief among these was the idea of personal responsibility. It goes without saying that most researchers are motivated both by a love of research and a hope that their research might make a difference in the world, particularly if they are focused on climate and environment. The idea of the university as a relatively neutral space for knowledge generation was also given as an ideal location from which to try to engage (see Box 1). So too was the idea of environmental activism, both mainstream and radical. Some researchers were explicit about the way their research deliberately aligns with their activist interests and gives them the opportunity to pursue these interests through advocacy work in ways that are politically engaged.

**Table:** 

**Box 1. LSE and the multiple motivations for engagement** Most researchers at LSE were interested in trying to influence events in the ‘real world’: ‘I feel that I’m dealing with an issue that is so important that I can no longer do research that is just interesting to me for the sake of it’. This led many to make the effort to engage with policy makers: ‘personally, I feel that my research in order for it to have the most impact needs to involve engagements with policy’. However, approaches to doing this varied. For some researchers, the main idea was simply to make efforts to communicate findings more effectively beyond the ivory tower: ‘I see my role as being in large part about creating research outputs, generating new knowledge, and then disseminating communicating, and interacting with people to use that knowledge in whatever way’. But for others the motivation was a more political one which recognised that the need to engage with power relations around knowledge production and exchange: ‘I see it as my role to highlight the issues that I see as being side-lined in discussions about climate change…’. Others were concerned to move even further beyond this insight to attempt more equal forms of co-production of knowledge and its application with local communities. Several wanted to try to use their privileged access to knowledge and policy processes that come with being part of a powerful university in the Global North to engage in knowledge brokerage, including speaking alongside and sometimes on behalf of those less powerful: ‘I have more legitimacy with certain of these people than they do and that means I have the opportunity to share perspectives that might not otherwise be heard’	

At the same time, there are range of pressures at play that help to drive engagement with policy. The first is the need for compliance with funder requirements. Many research funding bodies now require that careful attention is paid to what in the UK for example is termed ‘knowledge exchange and impact’ (KEI) and researchers are required to outline a strategy for achieving this as part of their research proposal. These include a range of funders ranging from Gates Foundation to the UK Economic and Social Research Council (ESRC), each with different definitions of impact, as for example set out by Wachbrit (2020). Partly as a result of this, there are also some expectations placed on researchers by universities themselves. It was reported that universities increasingly look favourably on researcher efforts to engage with policy. For example, Makerere University’s Centre for Climate Change Research and Innovations (MUCCRI) was established with this purpose in mind. It is one of the founding institutions, along with IUB’s International Centre for Climate Change and Development (ICCCAD), of the Least Developed Countries Universities Consortium (LUCCC). However, when it comes to individual researcher incentives and resources (such as career progression criteria), things are less clear, as discussed below.

Finally, there are market incentives that may drive researcher engagement. A key form of engagement was found to be applied consultancy work. Some researchers undertake consultancies as a means for topping up their incomes, especially in the Global South where academic salaries tend to be relatively low. Consultancies tend to be focused on forms of ‘applied’ research in which studies are commissioned with specific purposes in mind. While financial incentives were found to be common in both the North and the South, there were more nuanced implications in the latter e.g. in societies where resources are scarcer, applied research is prioritised by funders and conditionalities are strong, with the effect that adaptation discussions for example may tend to displace those about mitigation.

### How Do They Engage, and with Whom?

A range of different ways of engaging were reported, including conferences, policy round tables, formal and informal networking, and commissioned ‘applied’ research and consultancy. When asked about the different kinds of policy actors with whom interviewees engaged, we found that experiences were concentrated more with government and civil society than with the private sector. There was only a limited interest in, and understanding of, the multiple actors in policy landscapes, or an analysis of how policy processes operated. Among the Global South researchers, the world of policy was seen more sharply as different from (and even as hostile to) academia, and there was a stronger sense that to engage, as an academic, you had to learn and play by very different rules. Interviewees also tended to see the world of policy as distinct and largely separate from that of research. Despite the shortcomings discussed above, many still found linear models of the policy process to be a convenient shorthand way to conceptualize it, reflecting an earlier observation made by McGee (2004).

Conferences were one of the main ways that researchers tended to communicate their research findings beyond the university. These include academic conferences where policy makers may be in attendance, or policy events such as international meetings where climate policy issues are discussed by a range of stakeholders. One LSE interviewee was frustrated with these types of formal interactions because the same people tend to attend on the climate meetings ‘circuit’: *the danger with them is that you end up talking about the same things to same people, and I find it very circular.*

Policy roundtables were also common as an engagement tool. Most researchers had at some time participated in invited meetings by government policy makers interested in their views. However, in general government was seen as reluctant to commit the resources that would enable more engagement with researchers. In general, decision makers tended to see academics as too theoretically focused and detached from ‘real world’ issues. Reflecting on such events, an interviewee in Bangladesh remarked that university researchers needed to better understand the particular ‘mindset’ of policy makers in order to engage successfully. While this strategy was seen as a possible means to identify effective entry points, a resultant loss of autonomy and limits to critical thinking were also implied.

Related to both the above, researchers in all four universities noted the importance of networking to build connections and influence as key to engagement. They reported various formal opportunities to do this, such as academic conferences that are also attended by decision makers, as well as informal ones that rely on personal or informal professional connections. While many observed the usefulness of such informal networks, researchers in Bangladesh were also concerned about certain types of risk that they saw embodied in some personal networks (see Box 2).

**Table:** 

**Box 2. IUB, Bangladesh: strengths and weaknesses of informal policy linkages** Researchers in Bangladesh reported that informal links between academic and policy makers were particularly important and useful. One key insight was the idea that in addition to engagement, researchers also needed to ‘understand the pulse of the government’ as well, in order to understand and challenge dominant policy assumptions without losing the attention of trust of policy makers. For example, adaptation (rather than mitigation and low carbon development) is favoured, but informal discussions can help researchers to present ideas in ways that are acceptable but still challenge mainstream ideas. Researchers also pointed to the fact that a reliance on personal networks in Bangladesh was part of a wider problem of unaccountability and lack of transparency in public life. It was seen as an undesirable aspect of cultures of interaction in Bangladesh, as a ‘way of doing business’. Some interviewees were concerned about the dominance of highly personalised relationships and felt that it would be better to more fully institutionalise systems of more formalised relationships, such as regular consultation forums. As one interviewee pointed out, ‘everything depends on the government officer in place for a particular job’, and this creates risks around favouritism and discontinuity, since is not uncommon for officials to stay in post for relatively short periods of time. For this reason they were also distrustful of the idea of the knowledge broker as facilitator, in part because the word ‘broker’ in the Bangladesh context has negative connotations around the role of exploitative intermediaries acting as gatekeepers to public services and information	

As noted earlier, consultancy was discussed as a mode of policy engagement that in some cases enabled university faculty to adapt their existing scholarship and findings to an ‘applied’ context (e.g. LSE), and in others to gain access to research opportunities not easily available in a mainly teaching university setting (e.g. IUB). At both Makerere and IUB, where there is little university or public research funding, we found that most climate research took the form of consultancy research funded by development agencies.

However, several important shortcomings of consultancy research were observed, such as its ad hoc nature and the fact that it may give funders too much influence over research agendas. University-based researchers in Uganda also felt there were forced to compete with civil society organisations for such funding, which restricted collaboration. There was one unusual case at LSE of a productive long term consultancy collaboration between a private company and a researcher. In a programme of mutually agreed commissioned work over a period of several years both ‘applied’ and ‘pure’ research was embraced, even though the latter was primarily of interest only to the researcher. This was felt by both sides to ensure the production of high quality, in depth research as well as maintaining the researcher’s personal interest and avoiding ‘friction costs’.

As discussed earlier, some researchers reported taking part in training events aimed at enhancing decision makers’ knowledge and capacities. For example, training of government officials in relation to climate issues—which often draws on individuals’ research—was seen by as a useful way of indirectly engaging with policy by Makerere researchers. This was also reported in positive terms by researchers from IUB, where the International Centre for Climate Change and Development (ICCCAD) undertakes regular training courses.

Among some university faculty, teaching was viewed as an important indirect way to engage with policy. This is very much in line with the capacity building agenda set out in the Paris Agreement (Hoffmeister et al. 2016) but is rarely recognised as significant in discussions of university policy engagement. It was particularly emphasised in relation to masters level graduate students, where a future career often takes students into policy positions. For example, at LSE interviewees saw teaching as a mode of engagement that may shape future policy, and it was noted that maintaining links with former students can also lead to policy engagement opportunities. It was pointed out that the fact that students read academic papers and engage with their findings is not sufficiently recognised by those critical of the relevance of academic publications produced in the elite university setting. One interviewee remained sceptical that teaching produced policy influence, citing cases of students who had espoused radical ideas in class but who later became risk-averse once they had found their way into a professional position.

The use of research for advocacy and campaigning was reported as another way of engaging with policy, through adopting the role of what Pielke (2012) terms the ‘issue advocate’. However, this also went hand in hand with an awareness of political risk and concerns about objectivity. By contrast, the ‘discovery’ model of engagement, in which a researcher does not actively engage, but waits to be approached by a policy maker (or the media) soliciting a conversation or an assignment, was found to be quite common. As we have seen, in each of the universities, interviewees reported that most policy links were initiated by the policy maker, rather than by the researcher. Finally, there is still a constituency of conventional academic researchers who do not engage directly but hope that they can make a difference to the world simply through undertaking careful scholarship and teaching those who will in the future shape society.

On the question of engagement with different types of policy actors, informants reported that most of their links tended to be with governmental or inter-governmental actors. Many interviewees reported that they also had connections with non-governmental organizations (NGOs) or civil society organisations (CSOs), though these links were fewer among researchers in the Global South universities. One key gap in the design of the study was in relation to the media, about which we did not set out to collect information or experiences, apart from asking about the use of social media. In retrospect this was an oversight, as the media’s role in either shaping or undermining the public understanding of science is relevant to understanding the science-policy interface. For example, media was a theme mentioned by some of the IUB researchers in Bangladesh where television talk shows were felt to have sometimes proved useful ways for more prominent academics to engage in public debate with government policy makers.

One unexpected finding was the relatively low number of researchers who had a relationship with private sector actors. At first, we thought this might be because researchers assumed that if we were concerned with policy actors we must mean government, and that they did not think to report links with business organizations. But when we prompted further, we found that researchers often had no links with private sector organizations at all, other than in some cases to receive funding from private business foundations. One reason given for the lack of links in both Germany and Bangladesh was the view that profit-oriented companies were likely to be less interested in environment and sustainability issues. Another was a belief that businesses often required unrealistically short turnaround times with contract research. This relatively low level of engagement matters not only because there is a need to challenge business interest groups that have lobbied against taking action to combat climate crisis, but also because private sector innovation in technology has an important role to play in both mitigation and adaptation.

## Discussion: Beyond Linearity Towards multicausality and Fragmentation

A discourse of ‘evidence-based policy making’ (EBPM) has been in vogue since the 2000s, which portrays policy makers as being in search of rigorously obtained evidence on which to base their decisions. This idea has helped to focus efforts on systematising research evidence for policy, such as with the IPCC process. All this suggests that there is a renewed public appetite for finding ways to build closer links between research and policy. However, as we have seen, the critical literature on the research-policy interface leads us to question many of these assumptions.

First, the idea of EBPM has been criticised as naïve and ideological. This is partly because the nature of what is considered evidence is shaped by power relations, and partly because, as Young (2007, p. 1) has noted, research-based evidence in any case usually plays only a ‘very minor role’ in policy decisions, and that ‘policy makers will take more or less anything that can help them to make a decision which seems reasonable and has a clear message and is available at the right time’. Second, as Weiss (1982, p. 621) points out, academic studies rarely if ever offer the kinds of clear findings that can be directly acted upon by those making policy decisions. She is sympathetic to the disappointment that scientists often feel when their work is ignored, and instead makes the case for its indirect rather than direct relevance and effects. Researchers should continue to publicise their work and try to communicate their findings because this indirect contribution is still very important, since it ‘provides a background of data, empirical generalizations, and ideas that affect the way that policy makers think about problems’ (p. 621). In the light of this, the idea of ‘evidence-*informed* policy making’ (EIPM) is sometimes taken to be a more realistic goal than EBPM (Mayne et al. 2018).

Ansell and Geyer (2017) argu that rather than simply assuming that knowledge can be ‘harnessed to a model of top-down rational policymaking’, researchers need to recognise complexity and be more pragmatic, suggesting the value of a more contingent approach. For Mayne et al. (2018) ‘evidence matters, but its framing and the receptivity of policy-makers to its implications are as important as scientific assessments of its quality’ (p. 3). Furthermore, as Cairney (2016) points out, policy makers may prioritise the *relevance* of research evidence rather than its *quality* and may use ‘cognitive shortcuts’ to process and prioritise evidence in relation to the problems they are trying to solve.

Our data suggests that researchers often lack detailed or precise knowledge about the policy world, often regarding it simply as the ‘end point’ into which they try to project their findings, in a suitable form. This makes it even more difficult for researchers to access the ‘right’ opportunities to try to influence decision makers. To counter this, researchers may therefore need to build more sophisticated understandings and maps of the policy landscape itself as a complex arena of politics, institutions, resources, history and ideas, and not simply treat it as ‘an unproblematic given, without reference to the sociocultural contexts in which it is embedded’ (Wedel et al. 2005, p. 43).

As a result, approaches to knowledge exchange are becoming more sophisticated. Since the linear model of engagement inadequately reflects complex realities, including the fragmentation of policy processes, several other approaches have been explored alongside that of the ‘knowledge broker’ (Lightowler and Knight 2013). These include linking research and action (Lavis 2006), knowledge transfer arrangements (Lavis 2003), ‘evidence generation partnerships’ (Williamson et al 2019), ‘linkage and exchange’ arrangements (Lomas 2000), capacity building for ‘evidence-based health’ (Pappaioanou 2003), ‘research utilization’ studies (Elliott and Popay 2000), and research ‘co-production’ (Graham et al. 2019).

### Reflections on Academic Researcher Roles as Knowledge Brokers

The knowledge broker role nevertheless rests on the assumption that policy outcomes can be improved through the provision of scientific evidence. In the field of health, knowledge brokers are described as ‘faculty who are connected to policy makers as a conduit to policy influence and serve as advisors to academic peers on EIPM or Knowledge Translation’ (Jessani et al. 2016, p. 601). In practice, this is just one of many roles that a university-based faculty member or researcher might play, alongside carrying out research, teaching and academic administration, each requiring a specific skill set. The Climate Knowledge Brokers (CKB 2015) initiative identified the main potential knowledge brokerage roles as outreach, feedback, synthesising and filtering. Brokerage roles identified among our interviewees took various forms, including bridging between communities and policy makers, the co-production of research projects, and undertaking consultancy work.

However, we found that the knowledge broker role was not as widely recognised or adopted by individual researchers as we might have expected, particularly among researchers in the two Global South universities. In Bangladesh, as we saw in Box 2, the word ‘broker’ was not liked, since it has undertones of opportunism and exploitation and is associated with undesirable activities in wider society and politics. A second problem with the knowledge broker idea was the practical difficulty of performing the role, which requires complex unfamiliar skills and personal qualities—such as a certain outgoingness—that might not normally be associated with a person’s everyday research work. Also relevant here is the issue of the researcher life cycle, where an individual researcher may become recognised as a research leader or icon as their career develops, enabling them to take forward findings to policy more effectively. A third set of concerns was raised about ‘demand side’ problems, in which policy makers were perceived as unreceptive to researchers due to a different ‘mind set’ based on bureaucratic power and hierarchy. At Makerere’s MUCCRI, despite the unit being explicit about wanting to play a knowledge broker role (see http://muccri.mak.ac.ug/about), individual researchers were unenthusiastic about what they saw as the required ‘repackaging’ of their research findings, and the risk of merely performing engagement without much traction being achieved. Instead, some interviewees preferred to emphasise the importance of teaching as engagement and viewed their current students as important community outreach and policy actors in their future careers. The need for rethinking these issues is usefully illustrated by some at THK (Box 3).

**Table:** 

**Box 3. THK, knowledge brokering and North/South knowledge politics** Researchers expressed the idea that a university, particularly a technical one, is an independent space in which to construct knowledge objectively and build trust with policy makers. One researcher stated that [our] *‘general responsibility is to formulate socially relevant evidence and train new generation to tackle the challenges. The university is doing well but [it’s] not enough to solve all the problems’.* Others argued that universities in the Global North are nevertheless implicated in unequal global power relations. They argue that researchers, and the university itself, should commit more fully to knowledge brokerage activities with communities locally and internationally: *‘the university should not be trying to argue that we are neutral—that does not make sense because we are just a stakeholder like many others.’* The challenge of engaging in more socially relevant ways pushes the university into the difficult territory of trying to balance different North–South knowledge demands. One researcher said *‘our university—like many others—aims to play a growing local role in social innovation; however, when it comes to international cooperation our role is rather to bring in specialized knowledge or connect to international state of art’.* Another researcher pointed out the dichotomous nature of knowledge diffusion and brokerage dues to these uneven geographies. This dichotomy is amplified by the unequal power relations generated by the dominance of financial flows from the Global North: *‘[the] problem is that donors often predefine the thematic orientation of applied research projects—with [the result] that values could be imposed through science projects making a true co-design approach to research difficult.’* They went on to argue that new forms of engagement are needed to challenge this status quo, and that one of the ways to do that is for Northern researchers to listen more closely to researchers and community voices in the Global South	

This finding is consistent with evidence in the research and policy literature that suggests that knowledge brokerage can unhelpfully increase complexity and fragmentation in science-policy communication, and that in practice it can prove a far messier and more difficult process than literature suggests (Adelle 2015). Key barriers include ‘the apparent reluctance, at times, of the researchers to interact with policy makers at all’ and ‘the problem of competition between different researchers and research organisations to have “their” tools used in the policy making process’. Some of these issues are discussed in the following section.

### Opportunities and Constraints Around Engagements with Policy

Opportunities for university based researchers to engage with policy around climate crisis are both extensive and diverse. They may be driven by individual efforts to achieve personal satisfaction and add meaning to professional activities by increasing relevance, linking with activist agendas, or connecting with pressure for change beyond the university. Opportunities for engagement may be self-made, externally facilitated or university supported, or in many cases some combination of these factors. The majority of engagement experiences in our sample were self-made in the sense of researchers wanting to go the extra step above and beyond their normal research work to engage.

Researchers reported feelings of enhanced self-worth and enthusiasm as a result of taking their work out into the ‘real world’, from crossing boundaries of discipline, from combining research and practice, and from the satisfaction and ‘buzz’ of getting their work noticed and (possibly) used. In this sense, policy engagement was seen as productive in that it generated additional value in their professional and personal lives. For some researchers, the imperative to engage takes them in a mainstream direction, such as those who link with agencies engaged in international negotiations. For others, it implies more radical action and a critical approach to thinking about power and knowledge production: *I see it as my role to highlight the issues that I see as being side-lined in discussions about climate change… [and] to highlight the need for understanding that there are different perspectives on environmental change.*

Involvement in policy networks and conferences often relies on individual interest and initiative. Some researchers pointed out the challenges involved with this approach, such as interpersonal communication skills and personal qualities such as confidence. The potential problem with the fact that so much policy engagement is self-driven (including efforts by individual policy makers to reach out to particular researchers) is the risk of lack of consistency, weak levels of institutionalization, and limited sustainability of impact.

There were both individual level constraints (time, knowledge, skills, incentives) and institutional level constraints (support, resources, rewards, independence). Key barriers to policy engagement were a lack of suitable skills, a shortage of time and problems of politics. Research funders are increasingly demanding that more attention be paid to impact and engagement activities as part of the research outputs, yet this can lead to ritualised forms of engagement that may simply ‘performed’ to meet engagement criteria, without remaining sufficiently open to the opportunistic improvisational aspects of the more successful forms of policy engagement.

Some funders also require and even facilitate engagement activities, while policy makers sometimes approach researchers to learn from them, either by admitting them into ‘invited’ policy spaces, or by providing them with consultancy opportunities that draw on their research. While externally facilitated efforts to promote engagement can be useful, they also generate problems. For example, universities seeking to promote and support researcher engagement efforts and knowledge exchange increasingly support KEI activities through provision of resources and training, particularly in the Global North. However, some researchers perceived a lack of alignment in that policy engagement was not valued among the main criteria required for career progression.

While there were many common experiences, there were also some important differences between Global North and South (Box 4).

**Table:** 

**Box 4. Makerere University: multiple constraints in the Global South** In some respects the data from Makerere illustrates many of the common general difficulties faced by researchers across each of the four universities. The pressures of a heavy university teaching load mean that research time is valuable and limited, particularly for junior faculty, which leaves little scope for engaging with policy. Career progression criteria tend to give low priority to policy engagement work, and this creates further disincentives. When they do try to engage, researchers frequently find it difficult to initiate contacts with relevant decision makers, who are often busy, remote and difficult to engage. At the same time, Makerere highlights some of the structural inequalities more specifically faced by universities in the Global South that negatively affects policy engagement opportunities. Without much in the way of university support or training, researchers may lack skills and experience necessary to simplify and package research findings into ‘user-friendly products’. Only a few researchers with the right connections were able to approach and engage policy makers. It was also reported that government policy makers often have a negative view of universities as ‘ivory towers’ out of touch with their needs as decision makers. With relatively low levels of financial support available from the government, much of the climate research taking place is funded by foreign donors. This means that research agendas tend to be driven more by donor priorities than by local needs or country level plans. It also sometimes means that university based researchers may find themselves competing with civil society organizations for research opportunities. Some researchers felt that donors often prefer civil society groups to academic researchers as less rigid and more open to ‘short-term’ research assignments. Perhaps relatedly, some researchers also reported that they felt civil society organizations (CSOs) possess better skills and more time for policy engagement than academic researchers	

Our findings therefore suggest several key areas of *disjuncture* around the researcher/policy interface (Lewis and Mosse 2006a, b). The first is differences in the assumptions and practices found in these two social worlds. As Adil Najam has pointed out, the world of science is fundamentally at odds with that of policy in its values and basic orientation: ‘science is about understanding things; policy is about getting things done’ (One Earth 2020). The second is between the assumptions many researchers hold about the worlds of policy, and the realities of policy as a complex and diverse area of decision making, interest groups and contested meanings. Even the ostensibly straightforward category ‘policy maker’ (a common reference point for academics) lacks precision and tends to conflate different policy actors and levels—from politicians to administrators, and from senior decision makers to frontline implementers. Researchers may also assume that decision makers will act on research in good faith when in practice they may prefer to prioritise evidence that enables them to avoid taking responsibility for their decisions, as Kay and King (2020) have argued, or to be governed by short term priorities.^5^ In the Netflix produced satirical film *Don’t Look Up* (2021) Professor Randall Mindy’s character suggests that scientists can act as knowledge brokers but that their voices can quickly be co-opted by politicians to pursue business as usual and public pressure is required to drive political leaders to take action.^6^ A third area of disjuncture is the gap between researcher expectations about policy engagement and the level and type of support provided by their universities to help them in their efforts. Understanding these further may help explain why researcher efforts to engage with policy are often experienced as suboptimal.

The Paris Agreement identified the importance of capacity building for achieving its goals, yet this has yet to impact much on universities in terms of resources. Capacity building initiatives that *have* taken place tend to be Global North led and focused on Global South governments, relying on ‘fly-in fly-out’ approaches in which foreign consultants or international agency staff pay short visits to a targeted country to hold workshops or training sessions, involving presentations typically communicated in non-local languages (Huq and Nasir 2016). Unsurprisingly these encounters mainly fail to build meaningful institutional capacity in the longer term (Nakhooda 2015; Nasir et al. 2017).

Our data supports the need for a shift away from this model and towards supporting researchers, along with the universities of which they form a part, and particularly those in the Global South, to become more active partners in capacity building processes (Nasir et al. 2017). This will require further enhancement of the capacities of universities on both sides to build partnerships that are based on cooperation and exchange, rather than the traditional unequal model of Northern- and consultant-led capacity building (Huq and Nasir 2016).

## Conclusions and Recommendations

Our data provides insights into the diverse relationships that university based researchers may choose to pursue with policy makers, from active engagement to simply making their work ‘discoverable.’ In trying to make sense of these experiences we need to acknowledge this diversity and remain open to pluralism with regards disciplines, methods and scale. In general, these interactions are perceived as working best when they are based on clear communication, mutual trust and respect, where they have been built up over a long time, and where there is recognition of different actors’ operating mode and needs.

The analysis challenges certain still-dominant views of the relationship between research and policy. First, the idea that the research policy interface should be understood as a ‘gap’ that can be ‘bridged’ when researchers make their findings more easily available to policy makers is shown to be an oversimplification. Effective engagement instead depends on flexible roles, identities and subjectivities that require new forms of co-production, capacity construction and contestation rather than focusing simply on conveying findings to decision makers. The importance of building persuasive narratives, and not just presenting findings, will require researchers to develop new skills. Second, while we depend (partly for convenience) on working models that require us to keep research and policy in different compartments, we must recognise that they do not exist in separate spheres. They are subject to forms of boundary work that renders them shifting, intertwined and mutually constituted. Policies help to determine the subjects and types of research that are possible, the forms of research that are acceptable, and the terms of engagement by decision makers with findings. Policy impacts upon researcher choices, draws them into forms of engaged (or unengaged) identities, and allocates the resources that they seek to control through their professional activities. University researchers are therefore part of a web of policy relationships that they both help to construct, and are themselves partly constructed by, as they go about their work. Despite this, there is value in the idea that while the linear view of the policy process is at odds with reality it offers remains a useful shorthand [or ‘necessary fiction’, as McGee (2004) describes it].

Five principal conclusions emerge from the study:

*First*, research-policy interactions typically depend on individual efforts, not on institutional support. Many researchers feel, rightly or wrongly, that their universities provide insufficient resources or recognition for this. Engagement activities tend to be experienced by faculty in particular as additional burdens on top of the everyday core tasks of teaching and research. The challenge is to improve supporting structures on both sides of the science-policy interface—in the universities, and in the world of wider policy.

*Second*, despite its prevalence in fields such as public health, the idea of the ‘knowledge broker’ remains relatively underdeveloped in the climate field. This is true both in terms of limited researcher awareness of the ideas associated with knowledge broker roles, and of the possible types of knowledge broker activities that can be undertaken.

*Third*, climate researchers are most likely to engage with government, or with civil society, but less likely to engage with the private sector. They tend to focus on policy problem identification, but less with policy implementation or evaluation. They may not have a detailed understanding of how relevant areas of the policy world operate, or of the different actors and processes involved.

*Fourth*, while there is considerable diversity among researchers as to how they think about engagements with policy, relatively little attention is given to the potential for inter-disciplinary work, which is essential to informed action on climate change, or to research that is co-produced by universities with community level actors.

*Finally*, while it would be useful to draw standardised conclusions about the differences of experience among university researchers in the Global North and Global South, the high level of internal diversity *within* each university case in terms of individuals academics’ experiences does not sufficiently allow for this.

**Fig. 3:**
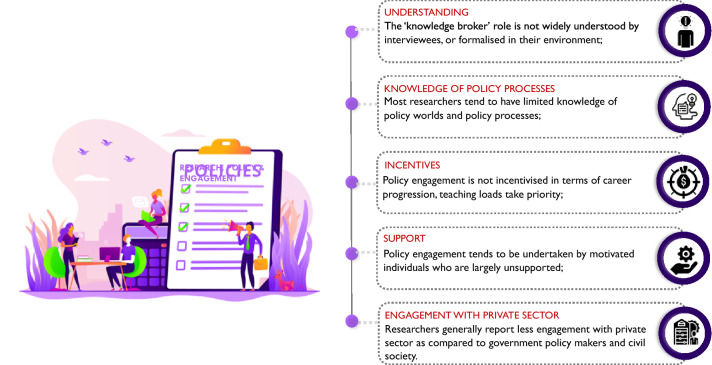
Common challenges among researchers

Four recommendations are made for improving the research-policy interface around climate research (Fig. 3):

1. *Support* Enhancing the institutional support available in universities and recognition for research engagement, both at the individual level, and through capacity building partnerships between universities and decision makers, as well as between universities in North and South (e.g. the ongoing funding scheme from the German Academic Exchange Service [DAAD] to establish collaboration between German universities and those in the Global South to support transformation to sustainability; the Development Leadership Programme at University of Birmingham that aims to foster coalitions between university researchers, think tanks, media, bureaucrats and politicians).
2. *Incentives* Creating incentives for developing initiatives that give more attention to multidisciplinary and cross-sectoral research approaches (e.g. a proposed Bangladesh-UK university research collaboration on climate crisis and development currently under discussion by some of the authors with the UK Foreign, Commonwealth and Development Office [FCDO]).
3. *Co-production* Giving more priority within universities to forms of research and teaching that encourage co-production of research agendas, both at community level, and with decision makers. This could include work where the researcher—as well as other knowledge intermediaries—plays broker roles that link community level interests to policy worlds, or bringing practitioners and decision makers into university teaching and training programmes (e.g. Manchester University’s partnership with Shack Dwellers International is designed to foster grassroots academic collaboration, and includes ‘community-led postgraduate teaching’ in which community leaders and practitioners deliver 60% of lectures, see Mitlin et al. 2019). The global rise of youth activism around climate issues is also an obvious area that calls out for deeper engagement by university based researchers.
4. *New tools* Identifying tools that can improve researcher awareness of policy worlds and enhance trust between researchers and policy makers (e.g. building long-term partnerships between researchers and policy makers; using secondments or placements to facilitate more understanding of each other’s opportunities and constraints; drawing on the post-Covid lessons around online communication as a cost effective and environmentally sustainable engagement tool). (e.g. a new planned collaboration between the Bangladesh Planning Ministry and Wageningen University to design a massive open online course (MOOC) to inform future generations of professionals and researchers about the Bangladesh Delta Plan 2100, a long term government initiative established in 2018 for managing the next 50–100 years).

There is no straightforward solution to the problem of building the ‘right’ environment for effective engagement between researchers and decision makers. The challenge needs to be shared—between universities on the one hand, and on the other, between the multiple stakeholders involved in policy worlds including the range of public, private and civic actors operating at local, national and international levels. More innovative institutional models will be needed to create spaces for cooperation and knowledge exchange. New resources will need to be committed. In short, if researchers are to become more effective at engagement, or at better utilising the services of knowledge brokers, we will need to think differently about these issues, including the structure and purpose of universities themselves.

## Annex: A Note on the Universities

IUB is one of Bangladesh’s first private universities, founded in 1993, and is mainly focused on providing undergraduate education. In addition to its teaching it has more recently begun to undertake research and develop global partnerships. In this regard, it is home to one of the country’s leading climate change research institutions in the form of the International Centre for Climate Change and Development (ICCCAD), established in 2010. ICCCAD seeks to influence environment and climate policy nationally and internationally, and has been instrumental in setting up research into policy networks such as the Least Developed Countries Universities Consortium on Climate Change (LUCCC). It also hosts the annual Gobeshona Conference, an international forum for climate change research, policy and practice. The researchers interviewed for the study were all connected with ICCCAD.

LSE is a specialised social science university. It has 23 departments, 16 specialised research centres and 3000 members of staff. It was ranked second in the world for social science research in the 2019 QS World University Rankings. There are around 12,000 students at LSE, just over half of whom are graduate students and two thirds of whom are international students. One of its research units is the Grantham Research Institute on Climate Change. The researchers interviewed included both university faculty and research staff, some of whom are on short term research contracts, at the Grantham Institute.

Makerere is Uganda’s leading public university and one of Africa’s oldest. It is at the forefront of research and teaching in the field of environment and climate change in the country mainly through its College of Agricultural and Environmental Sciences (CAES), and the Makerere University Centre for Climate Change Research and Innovations (MUCCRI), a research unit and think tank established in 2013. Some of its researchers have been able to provide inputs into some of Uganda’s national and international reports and plans. Makerere University became one of the founders of the Least Developed Countries Universities Consortium on Climate Change (LUCCC), a collaborative network for enhancing capacity of Global South universities to address climate change and build resilience. In this way, Makerere University recognises and seeks to play a ‘knowledge broker’ role in order to influence the ways public, private, and civil society actors collaborate nationally and globally to strengthen climate resilience.

TH Köln is a public education and research institution and is the largest applied science university in Germany. Much of the applied research undertaken is focused at the national and local levels with municipalities, but our interviews were conducted mainly with researchers at the university’s Institute for Technology and Resources Management in the Tropics and Subtropics (ITT). This means that the views of the informants may not apply to the university as a whole. ITT is an interdisciplinary unit focused on generating and applying knowledge about sustainability, technology and resource management issues, particularly in the Global South. It also seeks to build capacities of education, research and policy making through training and networking activities. It also hosts the Centre for Natural Resources and Development (CNRD) network.

## Footnotes

1 Some researchers recently took the radical step of calling for a moratorium on climate change research in order to draw attention to the need to expose and renegotiate this 'broken contract' between science and society (Glavovic et al. 2022).

2 A key problem is the lack of precision in the term ‘policy maker’. This mainly serves as an ‘othering’ term for researchers, but in practice covers a wide variety of decision makers.

3 None of the current international quality rankings used by universities to market themselves include metrics related to policy engagement. A radical new approach that does try to incorporate some kinds of engagement criteria has recently been proposed in a report by Douglas et al. (2020).

4 ‘We need a London School of Ecology and Economics’, *The Ecologist*, 11 January 2021. https://theecologist.org/2021/jan/11/we-need-london-school-ecology-and-economics

5 In electoral democracies the NIMTO effect—‘not in my term of office’—is a powerful disincentive to taking action on unpopular decisions that have long term rather than short term implications.

6 Broadening the ways climate research findings and issues are represented in order to communicate with an engage wider public audiences remains an underutilised approach by university based researchers. See for example Lewis et al.’s (2014, 2021) work on these issues in relation to novels, film, and music in the field of development; and Selim’s (2022) discussion of culture and climate struggles in Bangladesh.
